# Intra-ovarian injection of platelet-rich plasma into ovarian tissue promoted rejuvenation in the rat model of premature ovarian insufficiency and restored ovulation rate via angiogenesis modulation

**DOI:** 10.1186/s12958-020-00638-4

**Published:** 2020-08-05

**Authors:** Shahin Ahmadian, Sepideh Sheshpari, Mohammad Pazhang, Alberto Miranda Bedate, Rahim Beheshti, Mehran Mesgari Abbasi, Mohammad Nouri, Reza Rahbarghazi, Mahdi Mahdipour

**Affiliations:** 1 Women’s Reproductive Health Research Center, Tabriz University of Medical Sciences, Tabriz, 5138663134 Iran; 2 Department of Biology, Faculty of Basic Sciences, Azarbaijan Shahid Madani University, Tabriz, 537517169 Iran; 3 Department of Midwifery, Faculty of Nursing and Midwifery, Tabriz University of Medical Sciences, Tabriz, 5138947977 Iran; 4 Laboratory for Translational Immunology (LTI), Universitair Medisch Centrum Utrecht, (UMCU), Utrecht, Heidelberglaan 100, 3584 CX The Netherlands; 5 Department of Veterinary Science, Islamic Azad University Shabestar Branch, Shabestar, 5381637181 Iran; 6 Drug Applied Research Center, Tabriz University of Medical Sciences, Tabriz, Iran; 7 Stem Cell Research Center, Tabriz University of Medical Sciences, Tabriz, 5165665811 Iran; 8 Department of Reproductive Biology, Faculty of Advanced Medical Sciences, Tabriz University of Medical Sciences, Tabriz, 5166653431 Iran; 9 Department of Applied Cell Sciences, Faculty of Advanced Medical Sciences, Tabriz University of Medical Sciences, Tabriz, 5166653431 Iran

**Keywords:** Platelet-rich plasma, Ovarian rejuvenation, Premature ovarian insufficiency, Angiogenesis, Fertility

## Abstract

Premature Ovarian Insufficiency (POI) is viewed as a type of infertility in which the menopausal status occurs before the physiological age. Several therapeutic strategies have been introduced in clinic for POI treatment, although the outputs are not fully convincing. Platelet-rich plasma (PRP) is a unique blood product widely applied in regenerative medicine, which is based on the releasing of the growth factors present in platelets α-granules. In the current investigation, we examined the effectiveness of PRP as a therapeutic alternative for POI animals. POI in Wistar albino rats was induced by daily intraperitoneal (IP) administration of gonadotoxic chemical agent, 4-vinylcyclohexene dioxide (VCD) (160 mg/ kg) for 15 consecutive days. After POI induction, the PRP solution was directly injected intra-ovarian in two concentrations via a surgical intervention. Every two weeks post-injection, pathological changes were monitored in the ovaries using Hematoxylin-Eosin staining method, until eight weeks. Follicle Stimulating Hormone (FSH) content in serum was measured, together with the expression of the angiogenic-related transcripts *ANGPT2* and *KDR* by real-time qPCR. Furthermore the fertility status of the treated rats was evaluated by mating trials. Histopathological examination revealed successful POI induction via the depletion of morphologically normal follicles in rats following VCD treatment compared to the control rats. The injection of PRP at two concentrations reduced the number and extent of the follicular atresia and inflammatory responses (*p* < 0.05). The expression of both *ANGPT2* and *KDR* transcripts were significantly increased in POI rats due to enhanced inflammation, while these values were modulated after PRP administration (*p* < 0.05) compared to POI rats. FSH showed a decreased trend in concentration eight weeks after PRP treatment, but not statistically significant (*p* > 0.05). Nevertheless, a clear improvement in litter counts was found in POI rats receiving PRP compared to the non-treated POI group, being able to consider PRP as a facile, quick, accessible, safe and relatively cheap alternative therapeutic strategy to revert POI-related pathologies.

## Introduction

Women’s reproduction disability, mentioned also as infertility, is defined as the failure achieving pregnancy in a duration of 1 year with unprotected sexual intercourses [1]. Both male and female patients almost equally suffer from infertility complications [2], which are caused by different factors, such as genetic background, obstructive disorders, and environmental elements. It has also been well documented that chemicals or anti-cancer gonadotoxic drugs are major inducers of sub-fertility in females before the physiological age, known as premature ovarian insufficiency (POI). In this regard, most POI patients derived to the fertility clinics have previously received chemotherapy [3, 4]. Moreover, several complications are associated with POI such as osteoporosis, depression, cardiovascular diseases, etc. [3, 5, 6]. The routine treatment strategies are in general gonadotropin-releasing hormone agonist (GnRHa), hormonal replacement therapy (HRT), and assisted reproductive technologies (ART). These procedures, however, are not providing satisfactory recovery to the patients [7, 8]. For example, HRT-based treatments have been linked to undesired secondary effects, like breast and ovarian cancers, venous thromboembolism [9, 10], and incapability of reforming uterine volume and/or endometrial thickness [3, 11]. In the case of ART, which considered as the primary strategy applied for patients dealing with POI [12], several unwanted effects were usually observed, such as inconsistent clinical outcomes, invasive surgical procedures, or prolonged ovulation induction protocols [7, 13]. In consequence, a new generation of therapeutics based on certain cell and cell products are being developed to palliate these deficiencies.

Platelet-rich plasma (PRP) is a remarkable example. It is composed by a high density platelet concentrate, and has been typically used for the treatment of various problems, such as surgeries or diabetic wounds, osteoarthritis, skin, and soft tissue damages [14–18]. Platelets derived from megakaryocytes in the bone marrow are anucleated blood cells with a life span of 7 to 10 days in humans, and a bit shorter in mice [19]. They are easy to isolate and have an active role in wound healing and tissue repair due to their α-granules content [20, 21]. The α-granules contain more than 800 different proteins that have a paracrine effect on surrounding cells, especially on local mesenchymal stem cells (MSCs), promoting a rapid tissue regeneration [21].

Angiogenesis is a sophisticated biological phenomenon participating in physiological and pathological conditions, refers to the formation of de novo vascular units from the pre-existing vascular network. Importantly for POI reversal, the increase of vascularization in ovaries to provide an increase of blood nourishment, contributing to the acceleration of healing procedure and scavenging tissue debris [22]. The critical role of different ligands, notably angiopoietins (e.g. ANGPT1 or ANGPT2), and receptor tyrosine kinases (e.g. VEGFR-2 or KDR) has been well documented in relation to angiogenesis, and therefore we hypothesize that the measure of their expression after PRP administration in POI patients provides a good picture of the regeneration status [23].

The current investigation aimed to assess the effectiveness of PRP on ovarian rejuvenation in an experimentally 4-vinylcyclohexene dioxide (VCD) induced POI model in rats [24]. VCD, a side product of 4-Vinylcyclohexene (VCH), is usually employed as an adjuvant in epoxy resins; however it has been shown as well to selectively destruct both primordial and primary follicles [24–26], similarly to what occurs in POI. Therefore, after POI induction in rats with VCD, PRP solution was injected at two different concentrations into ovarian tissue. Subsequently, fertility status, follicular developments, and angiogenesis-related transcript expressions of *ANGPT2* and *KDR* were assessed using histological examination and transcriptomic assays. This study could help in addressing therapeutic alternatives in the restoration of ovarian tissue in POI patients.

## Materials and methods

### Animals

Eighty-six female Wistar albino rats aged between six to seven weeks were obtained from Med-Zist Co. (Tehran) and housed in a standard environment set for the temperature of 22 ± 2 °C and twelve hours of dark/light cycling with unlimited access to food and water. Rats were housed in polyester cages for 1 week for acclimation. All the experimental protocols were confirmed by the local committee of the ethics at Tabriz University of Medical Sciences (IR.TBZMED.REC.1396.876).

### Development of premature ovarian insufficiency (POI) model in rats

After a week of acclimation, rats were divided into two major groups of VCD and the control. The VCD group (*n* = 63) was intraperitoneally (IP) injected with 160 mg/kg VCD (Catalog number 94956; Sigma Aldrich) using an appropriate solvent for 15 consecutive days [27]. The rats in the control group (*n* = 18) received similar normal saline volume. After 15 days, three rats from each group were randomly sacrificed and serum and ovarian tissue were sampled to confirm the development of POI.

### PRP enrichment protocol

For this purpose, PRP kits (Catalog number 1019; Rooyagene Fertilize-Lympho) were used according to the instructions provided by the company. This kit was designated for isolation of PRP in different enrichment levels. In short, five healthy female rats (9–10 weeks old) were selected for blood sampling. To this end, rats were deeply anesthetized, and blood harvested directly from the heart using a syringe. The mixture of blood and anticoagulant were centrifuged at 2100 RPM for 10 mins at 22 °C. After supernatant transfer into the new tubes, the second centrifugation was performed at 4000 RPM for 6 mins at 22 °C. Next, the supernatant was discarded, and PRP retained. The total number of platelets in blood samples before enrichment was 3.3 × 10^5^/μL and, following enrichment, both low and high concentrated platelet densities raised to 8.5 × 10^5^/μL (PRP-a: 3-fold increase; 3X) and 21.6 × 10^5^/μL (PRP-b: 7-fold increase; 7X), respectively. PRPs were freshly injected into the ovaries.

### Intra-ovarian injection of PRP

**Fig. 1:**
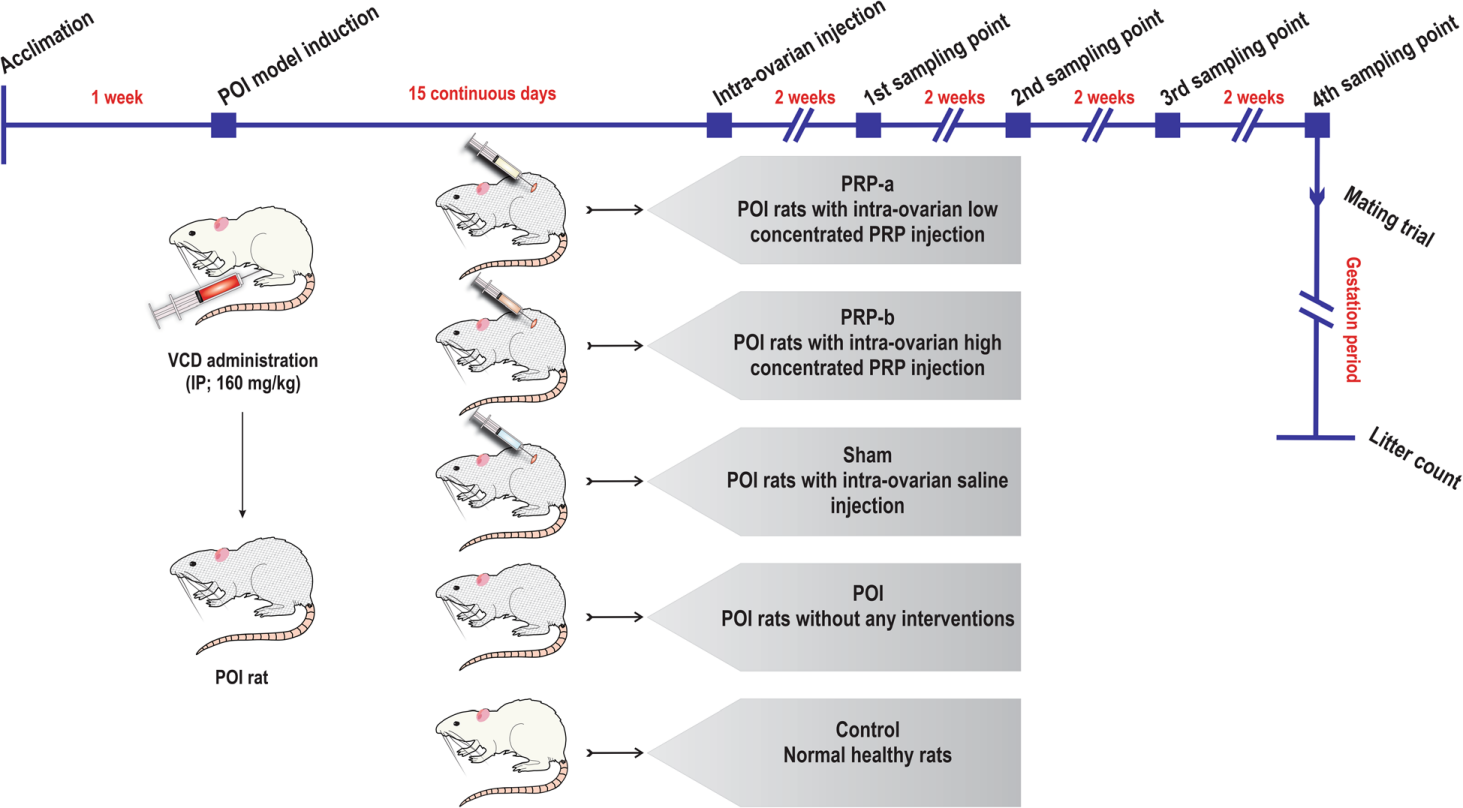
Timeline and groups before and after the PRP injection

After induction of POI, rats were randomly divided into four groups (*n* = 15 per group) consisting of (I): POI rats assigned for intra-ovarian injection of low concentrated PRP (**PRP-a**); (II): PRP rats with intra-ovarian injection of high concentrated PRP (**PRP-b**); (III): POI rats with intra-ovarian injection of normal saline (**sham**); and (IV): POI rats without any interference (**POI**). There were 15 rats in the control group without any intervention (Fig. 1).

**Fig. 2:**
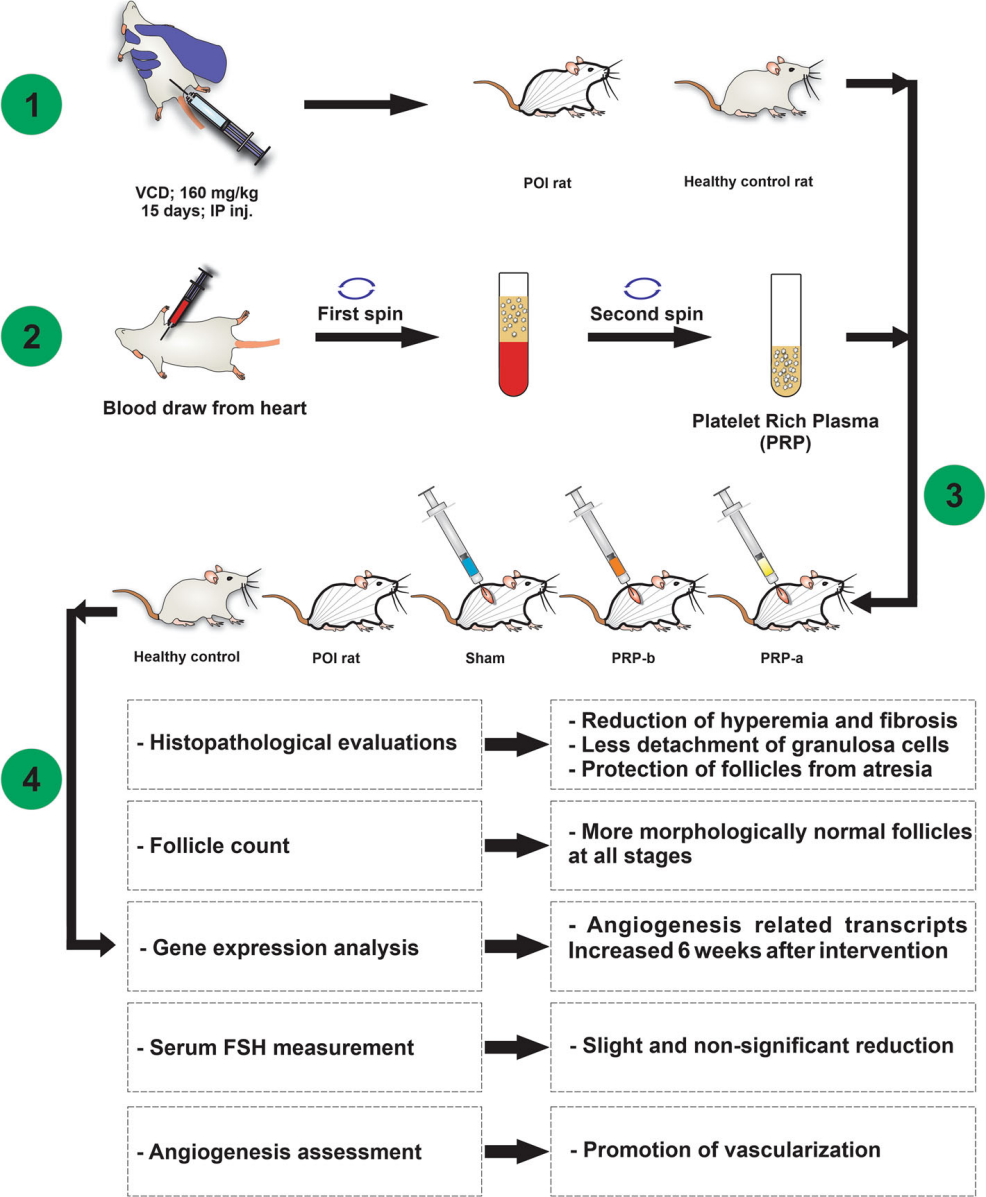
Graphical abstract of the summary of materials and methods. Animal model of POI rat was produced by IP injection of VCD (160 mg/kg) for 15 consecutive days **(1)**; blood collection followed by PRP enrichment **(2)**; intra-ovarian injection of PRP-a, PRP-b, and saline **(3)**; follow-up tests after intervention **(4)**

The surgical procedure was done following general anesthesia with a mixture of 90 mg/kg ketamine and 10 mg/kg xylazine solution. The day after the last VCD administration, the dorsum near to supra flank position was shaved and disinfected with 10% iodopovidone and 70% ethanol solutions. Subsequently, a small incision was made at both sides to access left and right ovaries. The ovarian injection was performed according to their groups’ specifications with freshly enriched PRP-a, PRP-b and, normal saline in the final volume of 10 μL using the insulin syringe (0.5 ml BD micro-fine plus 31G) (Fig. 2). In the end, the wounds were sutured and disinfected with an iodopovidone solution. No leakage was noticed during/after the procedure. Rats were supplied for five days, with 50 mg/kg of Neomycin to prevent bacterial infections.

### Sampling and histopathological studies

To perform the histopathological examination, ovarian samples were obtained every two weeks after PRP injection for two months. For this purpose, three rats were randomly selected per group. Rats were humanely euthanized (overdose of ketamine and xylazine) and left ovary was removed, rinsed in phosphate-buffered saline (PBS) solution, and fixed in 10% formalin (Merck). Right ovaries were sampled for gene expression evaluations and stored in liquid nitrogen until use.

For histopathological examination, the specimens were embedded in paraffin, and three consecutive sections of 5 μm thickness prepared using a microtome instrument (Leica). Then, the sections were stained with hematoxylin and eosin (H&E) staining solution. Any pathological response and structural changes were monitored in the ovarian sections. The numbers of primary, secondary, and antral follicles were recorded and compared with the control rats.

### RNA isolation, synthesis of cDNA, and quantitative real-time PCR

RNA extraction was performed using a Total RNA isolation Kit (YT9080, Yekta Tajhiz Azma; Tehran, Iran) according to the manufacture’s protocol. Extracted RNAs were reverse-transcribed by a cDNA synthesis kit (YT4500, Yekta Tajhiz Azma). Primer sets for *ANGPT2* and *KDR* transcripts (Table 1**)** were designed using NCBI online program (www.ncbi.nlm.nih.gov/tools/primer-blast/). Quantitative real-time PCR (qRT-PCR) reactions were carried out using synthesized cDNA samples and SYBR Green 2X (YT2551, Yekta Tajhiz Azma) in the “Roche LightCycler 96” instrument. Specific annealing temperatures were identified using gradient RT-PCR. PCR reactions were run on duplicates in three steps, basically consisting of denaturation, annealing and extension all for 10 s at 95, 60, and 72 °C, respectively over 40 cycles. The specificity of the PCR reactions was assessed by analyzing the melting curves.

**Table 1:** Primers sequences designed for Real-time PCR

Genes	Sequence (5′➔3′)		Annealing temperature (°C)	Product length
*Rn-ANGPT2* (*Rattus norvegicus angiopoietin 2*), NCBI accession number NM_134454.1	Forward	GCAGCGTTGACTTCCAGAGA	60	199
	Reverse	ATACAGAGAGTGTGCCTCGC		
*Rn-KDR* (*Rattus norvegicus kinase insert domain receptor*) NCBI accession number NM_013062.1	Forward	AGATGCGGGAAACTACACGG	60	184
	Reverse	GGGAGGGTTGGCATAGACTG		
*Rn-β-actin* (*Rattus norvegicus Beta Actin*) NCBI accession number NM_031144.3	Forward	TGACAGGATGCAGAAGGAGA	60	104
	Reverse	TAGAGCCACCAATCCACACA		

### Examination of vascular density using immunohistochemistry staining

To assess the angiogenic potential of PRP in the ovarian tissue post-VCD injection, we performed IHC staining of anti α-SMA (BIOCARE, CM 001 A) antibody. To this end, paraffin-embedded sections were used according to the previously published protocol [28]. In this study, we counted the number of α-SMA^+^ small arteries in 12 serial high-power field using light microscopy.

### Measuring serum levels of FSH using ELISA

To evaluate the possible correlations and feedback between serum levels of FSH and POI conditions before and after PRP treatment, we measured systemic FSH levels using an appropriate ELISA kit. For this purpose, blood samples were collected eight weeks after PRP injection. As mentioned above, blood samples were attained directly from the heart of the animals after a deep anesthetization using a mixture of 90 mg/kg ketamine and 10 mg/kg xylazine solution. Subsequently, serum samples were collected after centrifugation at 400×g for 20 min. All serum samples were stored at − 80 °C until use. ELISA was performed using a commercial kit (425–300 FSH AccuBind, CA, USA) according to the standard protocol provided by the company. This assay was performed in triplicate.

### Mating trial

To examine the fertility status of the rats, three remaining rats in all the groups were caged with fertility proved male rats in a ratio of 1:2 for five days. Afterward, every female rat was caged individually until the parturition, and the number of healthy litters per birth was registered.

### Statistical analysis

All the acquired results were provided in mean ± SD, and evaluated by GraphPad Prism 8. Data for each time-point after PRP injection were analyzed using One-Way ANOVA. To identify the significant difference between the groups, a post-hoc test (Fisher’s least significant difference, LSD) was applied. Statistical significance was set to *p* < 0.05.

## Results

### Rat model of POI was established after VCD administration

**Fig. 3:**
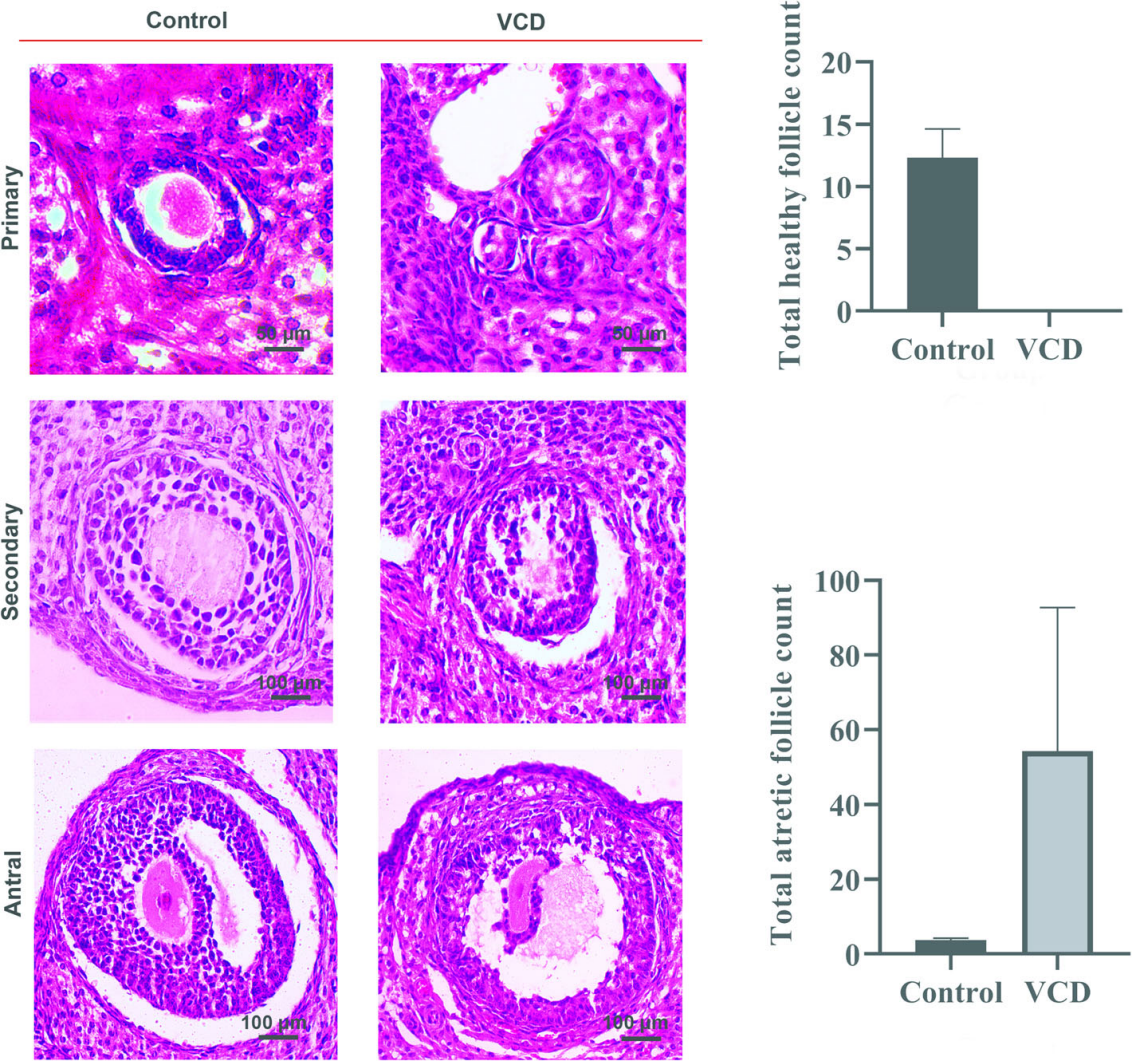
Confirmation of POI modelling using H&E staining. Microscopic imaging revealed both primary and advanced follicle stages in sections prepared from healthy ovaries. VCD administration promoted pathological changes in the structure of follicles indicated by atresia and shrinkage. All follicles, including primary secondary and antral stages, exhibited abnormal structure, detachment, disintegration, and degeneration of epithelial cells and granulosa cells in primary, secondary and antral follicles compared to the follicles from the control samples (**a**). Arrows = morphological alterations within the oocyte; arrow heads = detaching granulosa cells. The quantitative analysis revealed the depletion of morphologically normal follicles after VCD administration (*n* = 3). The number of atretic follicles was also increased after the 15-day injection of VCD **b**)

Following VCD administration for POI induction, histopathological evaluations revealed detachment of granulosa cells from oocytes, and general follicular atresia in all stages of follicular development including primary, secondary, and antral follicles (Fig. 3a). No morphologically normal follicles were noticed in the VCD rats in all stages of follicular development (average follicle number: Control = 12.33 ± 2.309; VCD = 0 ± 0) (Fig. 3b and Supplementary Fig. 1A). Accordingly, a larger number of atretic follicles were observed in the VCD group compared to the control rats (average follicle number: Control = 3.67 ± 0.58; VCD = 54.33 ± 38.40) (Fig. 3b and Supplementary Figs. 1B). These data confirmed that VCD can induce pathological changes in the ovarian stroma which leads to follicular atresia and depletion.

### Improved ovarian function was observed after PRP injection

**Fig. 4:**
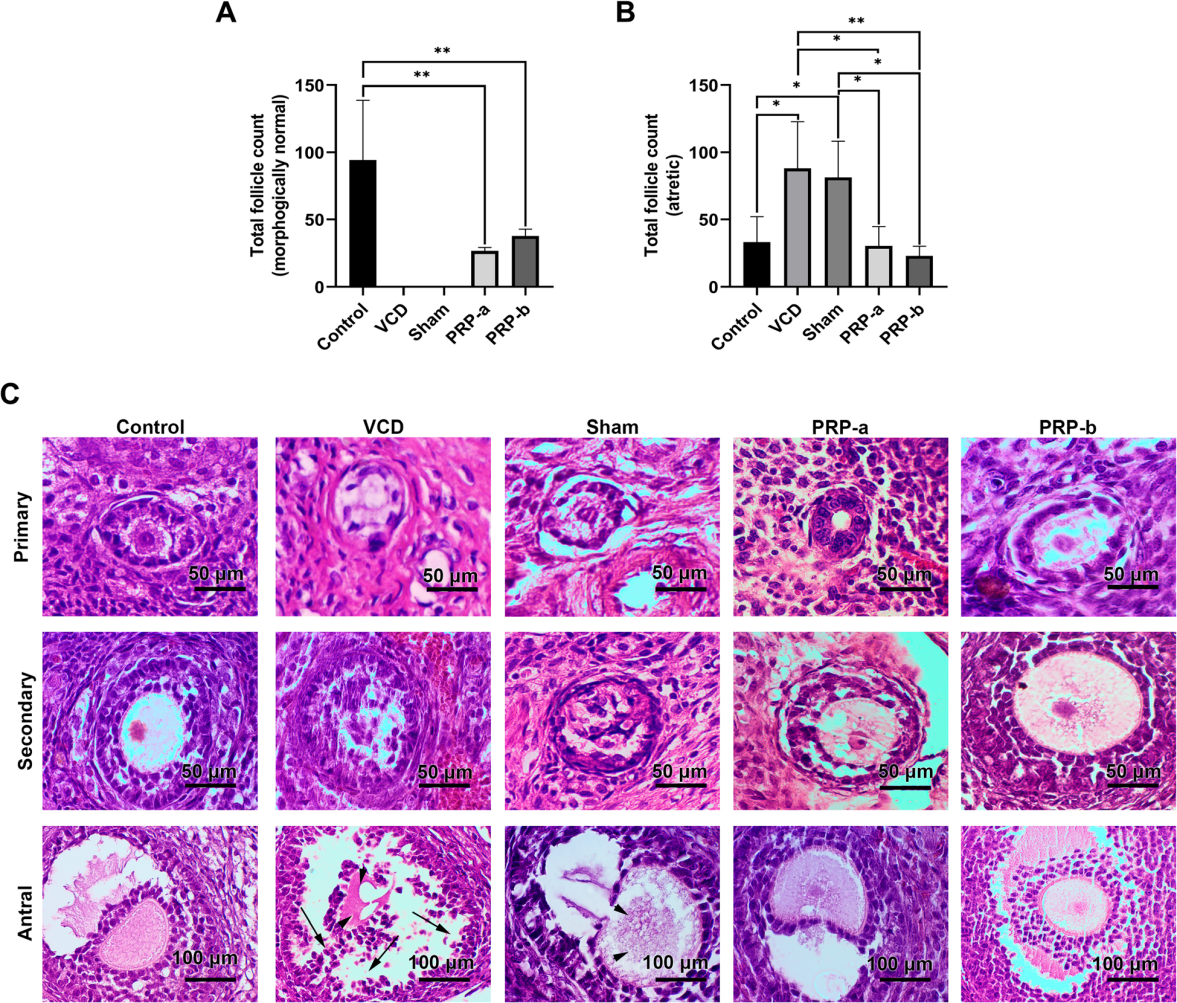
Follicle count after PRP administration. Total morphologically normal follicles count (**a**), and total atretic follicle counts (**b**) after intra-ovarian injection of PRP. One-Way ANOVA and LSD post-hoc analysis. *p < 0.05; and **p < 0.01 (n = 3). Bright-field imaging after H&E staining to visualize follicular status; 8 weeks after PRP treatment in all follicular stages of primary, secondary and antral. Arrows = detached granulosa cells; arrow heads = degenerating oocytes (**c**)

Histopathological studies demonstrated that rats which received PRP treatments in both concentrations exhibited general improvement in follicular quality, and statistically significant increases in total morphologically normal follicular counts when compared to the POI rats (Control = 94.33 ± 44.46; VCD = 0 ± 0; sham = 0 ± 0; PRP-a = 26.67 ± 2.517; PRP-b = 37.67 ± 5.132) (Fig. 4a-c**)**. In the POI and sham groups however, no morphologically normal follicles of any stages were noticed whereas, a significantly higher ratio (*p* < 0.05) of total atretic follicles were detected in these groups (Control = 33.00 ± 19.00; VCD = 88.00 ± 34.77; sham = 81.33 ± 27.02; PRP-a = 30.33 ± 14.36; PRP-b = 23.00 ± 7.00). Furthermore, a significant (*p* < 0.05) decrease in the atretic follicle count was observed in rats that received PRP compared to the POI and sham groups (Fig. 4a-b and Supplementary Fig. 2). The mean follicle count (the mean of all sampling points) in different groups after the intervention are presented in Supplementary Table 1. In addition, we noted that VCD injection resulted in pathological remodeling (fibrosis) and hyperemia, indicated by dilated vessel structures. Interestingly, following PRP injection at both doses, these features were blunted (Supplementary Fig. 3).

### PRP injection influenced expression of genes involved in angiogenesis

**Fig. 5:**
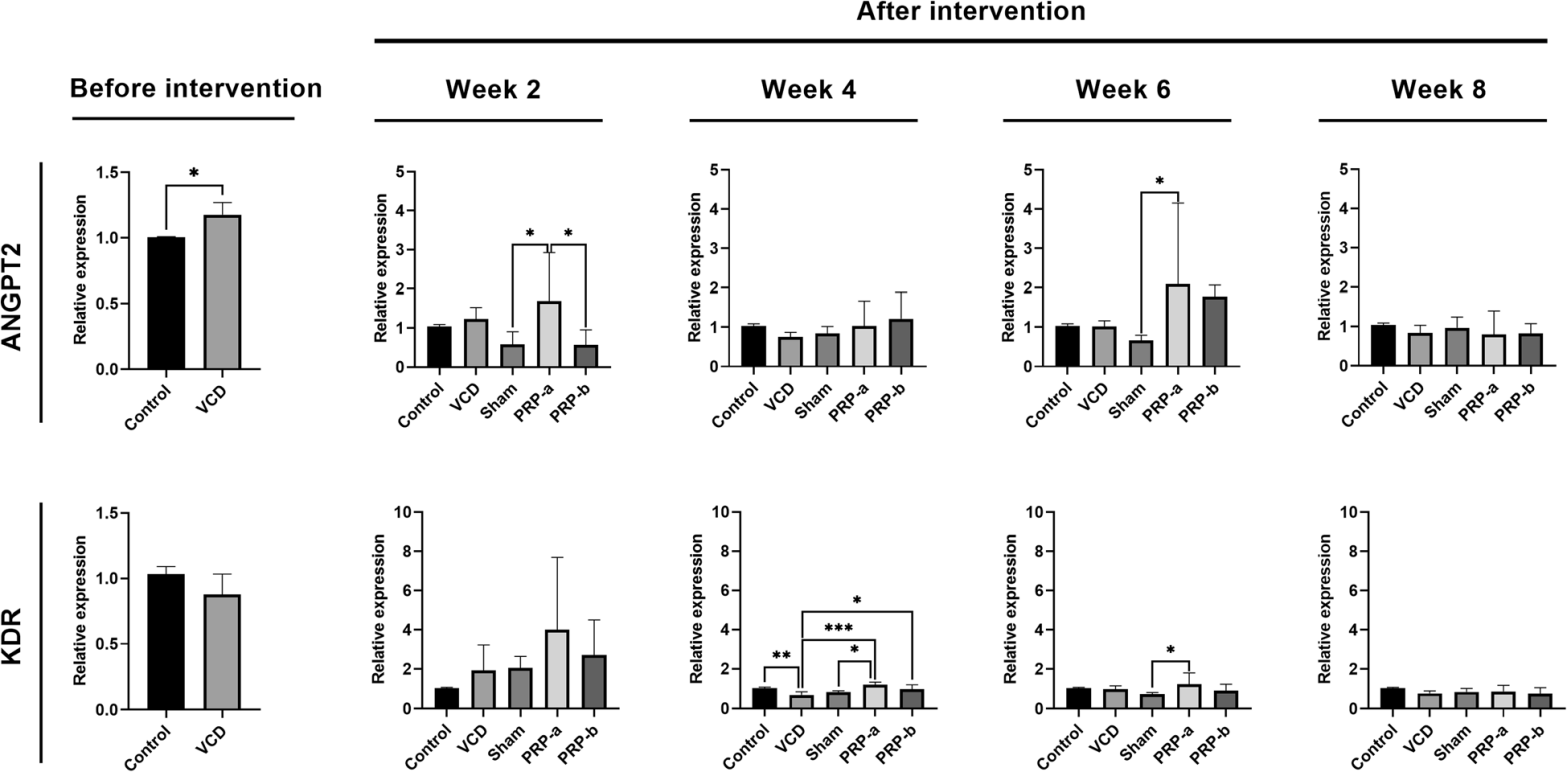
Relative expression of *ANGPT2* and *KDR* genes before and after PRP administration. One-Way ANOVA and LSD post-hoc analysis. *p < 0.05; **p < 0.01; and ***p < 0.001 (n = 3)

Real-time PCR analysis revealed that the transcript level of *ANGPT2* was increased after VCD administration, whereas *KDR* expression did not change significantly compared to the control group (*p* < 0.05; Fig. 5). Following PRP injection, a higher expression of both transcripts of *ANGPT2* and *KDR* were noticed in both PRP groups compared to other groups (*p* < 0.05), showing the highest levels on the 6th week after PRP injection in both PRP-a, and PRP-b groups (Fig. 5). At the latest sampling time point, however, the gene expressions have reached the plateau levels for both *ANGPT2* and *KDR* (Fig. 5).

### PRP induced ovarian tissue vascularization via the increase of α-SMA^+^ vascular units

**Fig. 6:**
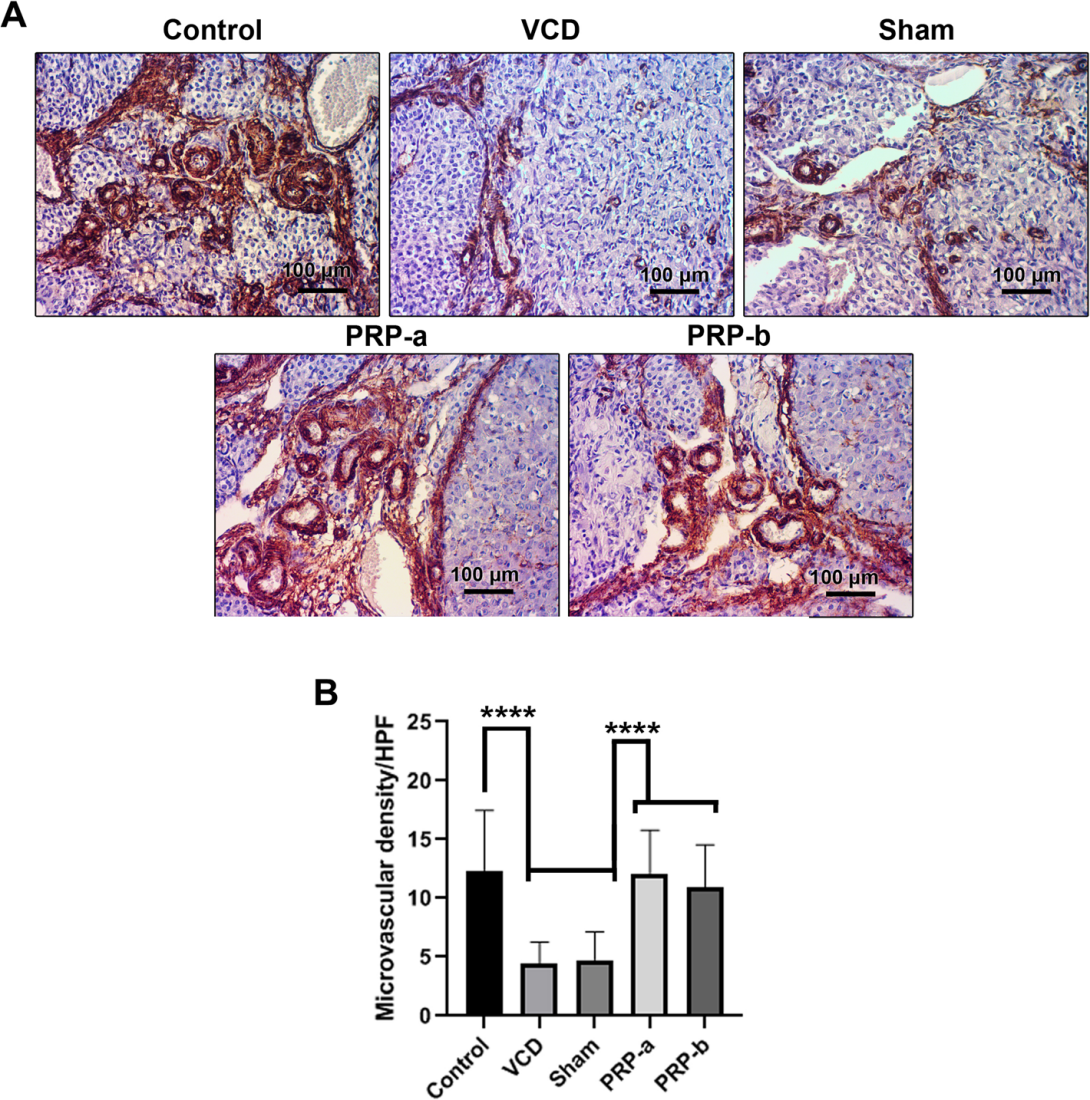
IHC staining for α-SMA^+^ small arteries to assess angiogenic potential of PRP-a and PRP-b (**a**), quantification of vascular density/HPF (**b**), 8 weeks after intervention. One-Way ANOVA and LSD post-hoc analysis. ****P ≤ 0.0001

IHC imaging revealed that the number of α-SMA^+^ vascular units was decreased in VCD and sham groups compared to the control group (*p* < 0.0001: Figure A-B). The injection of PRP in two different doses decreased the inhibitory effect of VCD and increased the vascular density, since 8 weeks after treatment the number of α-SMA^+^ small arteries was augmented in PRP-treated groups compared to the VCD and sham groups (*p* < 0.0001: Fig. 6a-b). No statistically significant differences were detected between the control group and PRP-treated rats in terms of α-SMA^+^ small arteries. These data showed that the application of PRP in VCD-treated rats could reduce the inhibitory effect of VCD on ovarian tissue vascularization.

### PRP injection did not affect serum levels of FSH

**Fig. 7:**
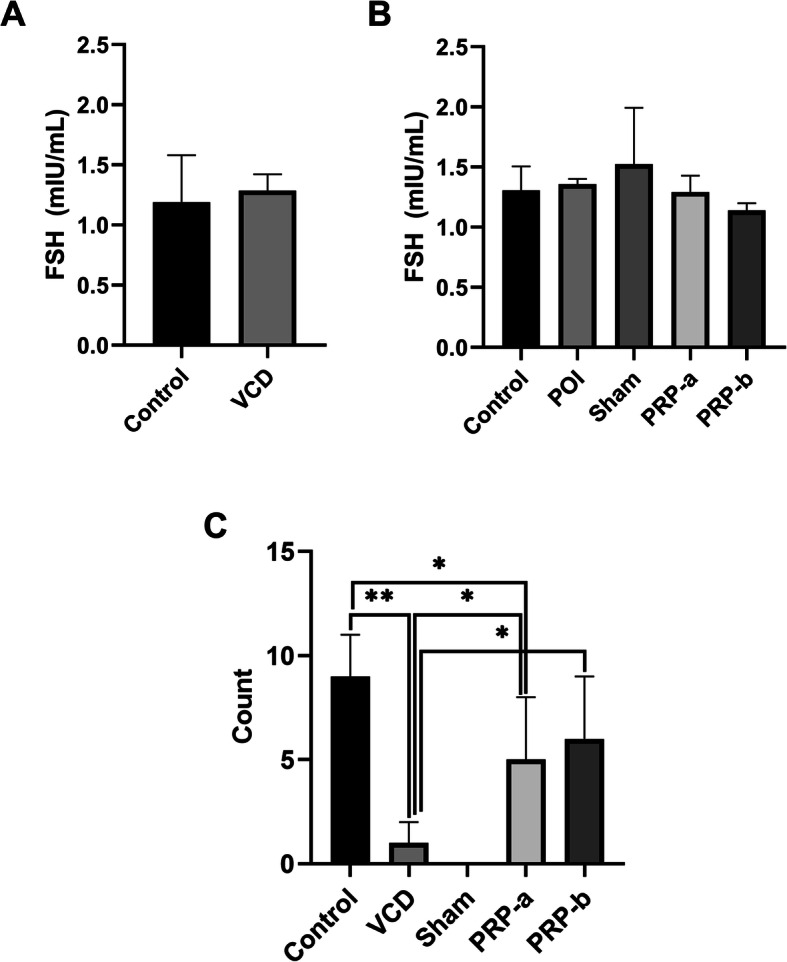
Serum levels of FSH before (**a**) and after (**b**) intra-ovarian injection of PRP. Mating trials following PRP treatment 8 weeks after intervention. The number of healthy litters per birth was stated (“y” axes) in all of the groups of the study (“x” axes). One-Way ANOVA and LSD post-hoc analysis. *p < 0.05; and **p < 0.01 (n = 3)

15 days after VCD administration, a mild increase of FSH level was observed in VCD group compared to the control rats; however, the difference was not statistically significant (Fig. 7a). Eight weeks after the PRP intervention, a declining pattern of FSH levels in the PRP treated versus non-treated rats was shown, although again not statistically significant (Fig. 7b). We further noticed that the lowest FSH levels were observed in PRP-b rats.

### Intra-ovarian injection of PRP improved fertility status of POI rats

To evaluate the fertility status of the rats after PRP injection, eight weeks after surgery mating trials were performed in 3 rats per experimental group. It was observed that the rats in the both PRP groups have restored their fertility status (average number of litters/birth: PRP-a = 5 and PRP-b = 6), moving closer to the healthy control rats (average number of litters/birth: 9). The results were significantly higher (*p* < 0.05) than POI and sham groups (average number of litters/birth = 1 and 0, respectively) (Fig. 7c).

## Discussion

It has been revealed that general clinical practices such as HRT, ART, and GnRHa, are not effective enough for POI conditions [3, 7–11]. Nowadays, several alternative therapeutic approaches, such as cryopreservation and stem cell therapies, are at the new front line [29, 30], but to determine the most beneficial ones, several advancements have been made in the field of animal modeling.

Mayer and colleagues in 2004 reported that daily IP injection of VCD (160 mg/kg) for 15 continuous days is an optimum dose to induce POI conditions, significantly diminishing the population of morphologically normal follicles within the ovaries [27]. In this study, POI physio-pathological features were achieved, like follicular depletion and atresia. From the therapeutic perspective, animal models have also been successfully employed, like in the work of Dehghani et al., in 2018. They reported that IP injection of PRP in POI rats increased the healthy primary and antral follicle counts, and they pointed out PRP as a protective agent against ovotoxic chemicals [31]. Along with the conduction of experiments involving intra-ovarian injection of PRP in animal models, some clinical trials have been started in human, opening hopes and therapeutic avenues in ovarian regeneration [32, 33].

Takehara and colleagues reported that administration of adipose derived mesenchymal stem cells (ADMSCs) could increase morphologically normal follicles counts in the treated rats [34]. In that line, our observations revealed that following intra-ovarian injection of PRP in both low and high concentrations, morphologically normal follicles were almost restored in all stages of follicular development, virtually reaching healthy control counts. As expected, no healthy primary follicles were observed in the POI rats. Evaluating the number of morphologically normal follicles (secondary and antral), there was a significant increase in the PRP-treated rats group versus the POI control. These observations illustrated the efficacy of PRP for the regeneration of ovarian tissue, possibly by shuttling growth factors into the site of injury [35]. Along with these changes, the count of atretic follicles was also obtained in a declining trend for PRP-treated as compared with the POI rats. In contrast to morphologically normal follicles, however, while atretic follicles were significantly reduced in PRP-a administered rats, a higher level of atresia was notified in the PRP-b group. This value could be due to the follicular dominance selection and negative feedback on other growing follicles leading the atresia [36]. Remarkably, the existence of different growth factors inside the platelet-derived granules could revert the cellular damage by increasing rejuvenation phenomenon [37]. Therefore, it is tempting to speculate that the increase of follicular reservoir in PRP-treated rats correlates with the inhibition of VCD-induced injuries.

In 2019, Ling and colleagues reported that human amnion-derived mesenchymal stem cells (hAD-MSCs) administration in POI rats caused overexpression of *Bcl-2* and *KDR* transcript levels [38]. These findings probably were due to the decrease in apoptosis rates and the promotion of angiogenesis within the damaged organs, as well as regulation of follicular development and ovarian function [38]. In the abovementioned work of Takehara and colleagues, the transplantation of ADMSCs to POI rats also increased the gene expression levels of *KDR* and *IGF-I* compared to the non-treated groups, promoting angiogenesis rate and ovarian restoration [34]. In addition, Yao and co-workers reported that chemotherapy down-regulated the expression of *VEGFR1* and *KDR* transcript levels, while human amnion epithelial cells (hAECs) transplantation into POI mice contributed to their up-regulation, likely by promoting pro-angiogenic signaling pathways [39]. In our study, VCD administration significantly elevated the transcript levels of *ANGPT2* compared to healthy control rats, probably due to the inflammation caused by the chemical itself [40]. Higher expression levels of *ANGPT2* and *KDR* were noticed in both PRP-a, and PRP-b treated groups compared to the POI rats, consistent with the increase of α-SMA^+^ small arteries in IHC imaging [37]. These results possibly indicate that the regeneration of POI ovarian tissue is done through the activation of pro-angiogenic pathways. Accordingly, some works have already highlighted the potent angiogenic activity of platelets via the secretion of, between others, SDF-1a, bFGF, VEGF and IL-8 [41–43]. The highest expression levels in both treatment groups were 6 weeks after PRP administration, and in the subsequent weeks, a plateau pattern was reached. This could probably be due to the fact that the optimal concentration of PRP and a balanced growth factor delivery were reached [44–46]. These findings illustrate that in the long term periods, the healing properties of PRP could diminish or, alternatively, that the stable conditions required for regeneration are possibly achieved. These data coincide with the recent report of Sfakianoudis and colleagues, in which six weeks after the autologous PRP treatment, a significant decline in serum FSH levels in the premature menopause patients were recorded [47].

Regarding the evaluation of fertility status after the PRP injection, the mating trial retrieved a significantly higher number of healthy litters in both PRP-treated groups compared to the non-treated ones. In agreement with our findings, and having stem cell infusion as the more recent gold standard [34], Mohamed and colleagues transplanted umbilical cord blood mesenchymal stem cells (UCMSCs) to POI mice, getting a higher number of born litters than in the untreated POI group [48]. Similar results were obtained as well from Lai and co-workers in 2014 and Su and colleagues in 2016, in where skin derived mesenchymal stem cells (SMSCs) and ADMSCs combined with collagen scaffold were used, respectively [8, 49]. Several other reports confirmed as well the effectiveness of cell therapy on fertility restoration and litter counts [7, 34, 49].

Analyzing the FSH hormonal status after intervention, it revealed a minor decline in treated versus non-treated rats, which was not statistically significant. The possible reason of these little differences could be the selective blocking effect of VCD on the c-Kit receptor (a tyrosine kinase receptor) in oocytes [24]. In fact, the main ligand of c-Kit (KITLG), which is a type of growth factor produced by granulosa cells, has an essential role in follicular survival [24]. In a recently published article, Carolino and colleagues studied the endocrine profile of POI rats induced by VCD. They reported that the FSH levels in POI rats did not significantly change for at least 90 days after the VCD administration [50]. In another study, VCD did not influence the FSH levels until 240 days from the start of the experiment [24], and Mayer and colleagues reported that VCD injection to mice do not significantly increase the FSH levels until 37 days [27]. Taking everything together, it could be concluded that an eight-week follow up might be not a long enough period to alter FSH levels in an animal model of POI induced by VCD.

Despite the superior effect of PRP at higher concentration compared to the low concentration in terms of follicle count in almost all time points, litter counts following mating, FSH serum levels and atretic follicle numbers, were not statistically different between the groups. In terms of gene expression analysis, although not statistically significant, PRP-a treated rats exhibited better results, probably due to the negative feedback from a high concentration of platelets in the PRP-b treated subjects. These findings highlight the critical role of PRP concentration in the modulation of angiogenesis signaling pathway within the injured ovaries.

Nowadays, several clinical centers have initiated PRP therapies for various reproductive-related complications, including POI and poor responding patients. As a clear advantage, the PRP-therapy can easily be used as co-adjuvant in patients undergoing chemotherapy, which are already being treated with other fertility procedures, such as ovarian tissue preservation. Additionally, PRP therapy possesses some interesting improvements that surpasses the benefits of other current cell sources for transplants. For instance, derived from its autologous origin, PRP administration does not induce auto-immune response after transplant, a highly prevalent and detrimental secondary effect of the actual therapies. Another example could be that, due to the existence of numerous granules, the introduction of PRP provides a high concentration of growth factors and regenerative modulators directly into the injured sites [18]. Consequently, it is noteworthy to highlight this cell source as a powerful healing product, which can potentially be used in a wide range of pathological processes like, between others, the POI.

Talking about suitable clinical settings to administrate PRP, transvaginal ultrasound- guided injection is a good candidate, since it is a nonsurgical technique that allows the clinicians to inject biological products like cells/PRP directly into the ovarian tissue. This technique is performed under sedation and is basically the same method used for an egg aspiration/retrieval during IVF/ICSI operation [32, 51]. As described above, stem cell transplantation is another putative therapeutic option for POI patients and potentially more powerful in combination with PRP; however, stem cell therapy is sometimes associated with tumorigenesis, making this approach less safe than others [52]. Therefore, PRP treatment could be listed as one of the least invasive procedures, even though, all the possible complications should be evaluated carefully in the future works.

## Conclusion

We have demonstrated that PRP can partially restore the function of POI ovaries. It can protect morphologically normal follicles from degeneration and atresia caused by ovotoxic chemicals and stimulate angiogenesis. Moreover, PRP showed a good capacity for tissue rejuvenation, inflammation regulation, minimizing the histopathological damages, and promoting the litter counts. Importantly, the PRP concentration has an important effect on the therapeutic outcome of PRP administration. If excessive, PRP will reduce its efficacy due to possibly a negative feedback generated by its components. It is therefore needed to find an optimized dose for PRP in any specific application to gain the best outcomes. Besides, PRP therapy is relatively cheap, safe, and does not require complicated procedures for its administration thus, overall, PRP could be considered as a putative alternative strategy for a variety of complications linked with female infertility, including POI.

## Supplementary information

**Additional file 1: Supplementary Figure 1.** Separate follicle counts before intra-ovarian RPR injection (*N* = 3), morphologically normal follicles **(A)**, and atretic follicles **(B)**.

**Additional file 2: Supplementary Figure 2.** Detailed morphologically normal and atretic primary, secondary and antral follicle count; 2, 4, 6 and 8 weeks after intra-ovarian PRP injection. One-Way ANOVA and LSD post-hoc analysis. *p < 0.05; **p < 0.01; and ***p < 0.001 (*n* = 3).

**Additional file 3: Supplementary Figure 3.** Bright-field imaging after H&E staining to visualize hyperemia and vessel dilation **(A)**, and fibrotic changes **(B)**, following the administration of PRP in VCD-treated rats on 8th week.

**Additional file 4: Supplementary Table 1.** Mean follicle count in different groups after the intervention (mean ± SD).

## Data Availability

All data generated or analyzed during this study are included in this published article and its supplementary information files.
