# Supplementary figures and images for: Intra-ovarian injection of platelet-rich plasma into ovarian tissue promoted rejuvenation in the rat model of premature ovarian insufficiency and restored ovulation rate via angiogenesis modulation
Source: Reprod Biol Endocrinol. 2020 Aug 5;18:78. doi: 10.1186/s12958-020-00638-4 (PMC7405361; doi:10.1186/s12958-020-00638-4)

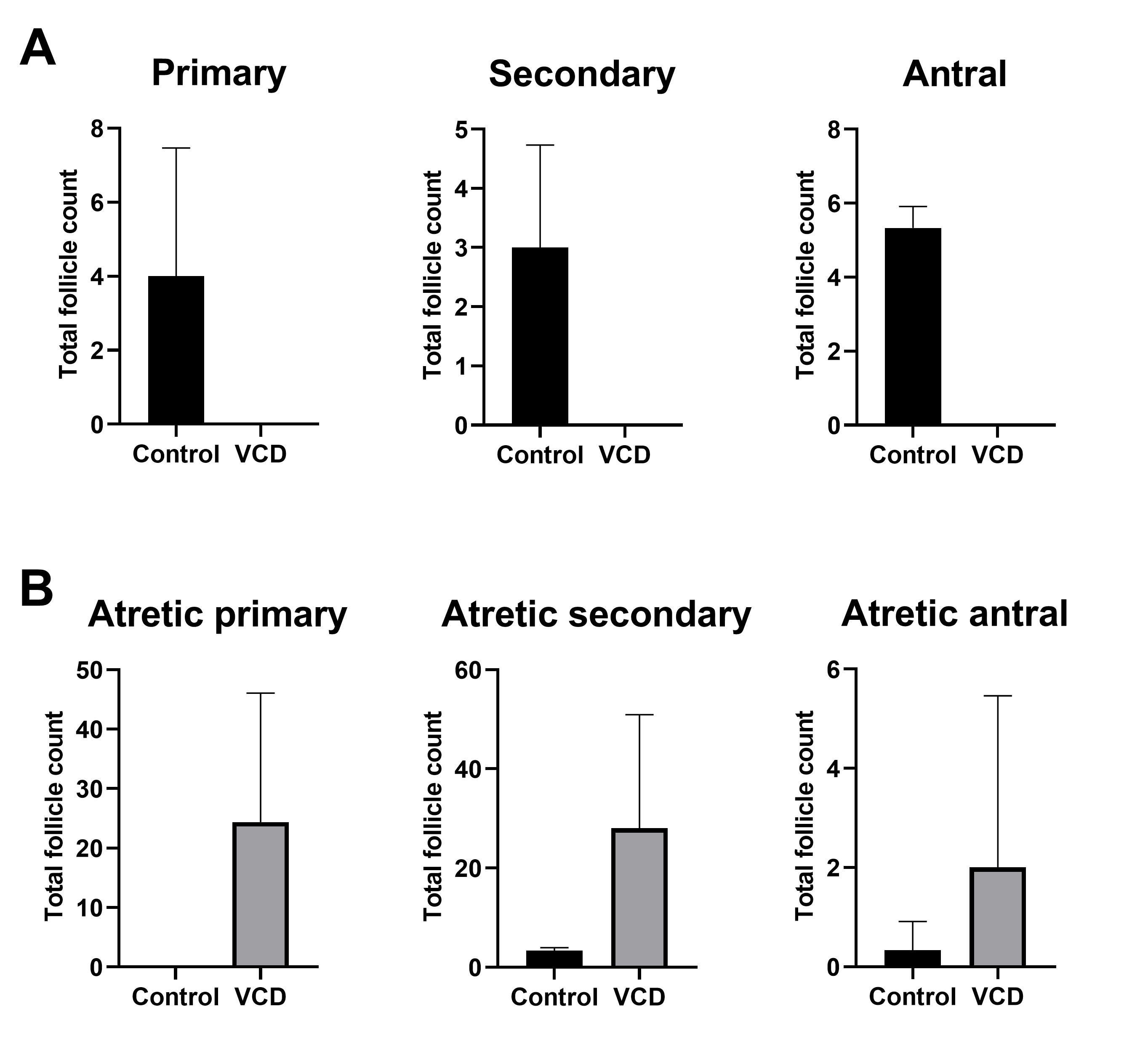

Supplement: Supplementary file 1 — Additional file 1: Supplementary Figure 1. Separate follicle counts before intra-ovarian RPR injection (N = 3), morphologically normal follicles (A), and atretic follicles (B). [file 12958_2020_638_MOESM1_ESM.tif]

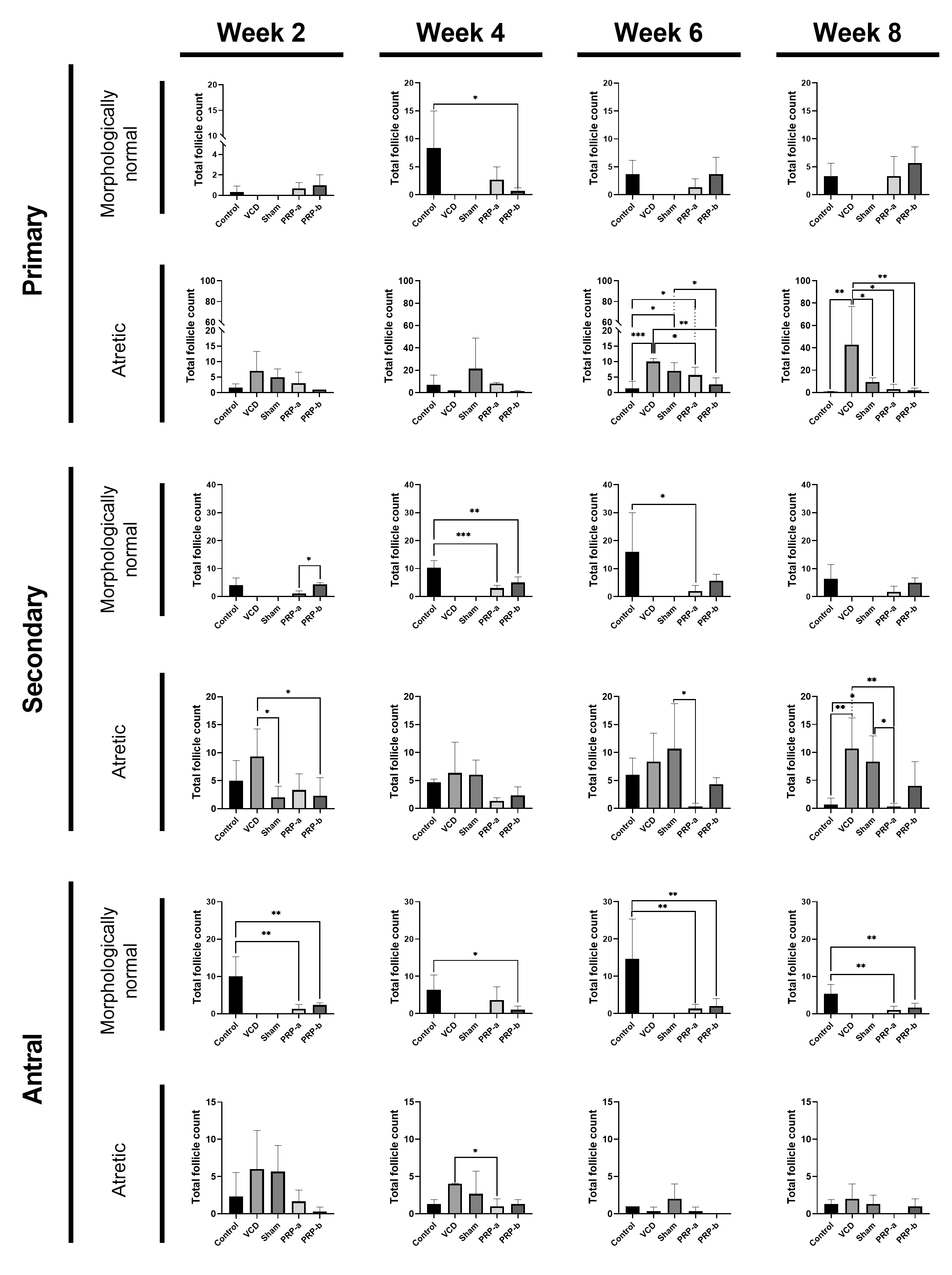

Supplement: Supplementary file 2 — Additional file 2: Supplementary Figure 2. Detailed morphologically normal and atretic primary, secondary and antral follicle count; 2, 4, 6 and 8 weeks after intra-ovarian PRP injection. One-Way ANOVA and LSD post-hoc analysis. *p < 0.05; **p < 0.01; and ***p < 0.001 (n = 3). [file 12958_2020_638_MOESM2_ESM.tif]

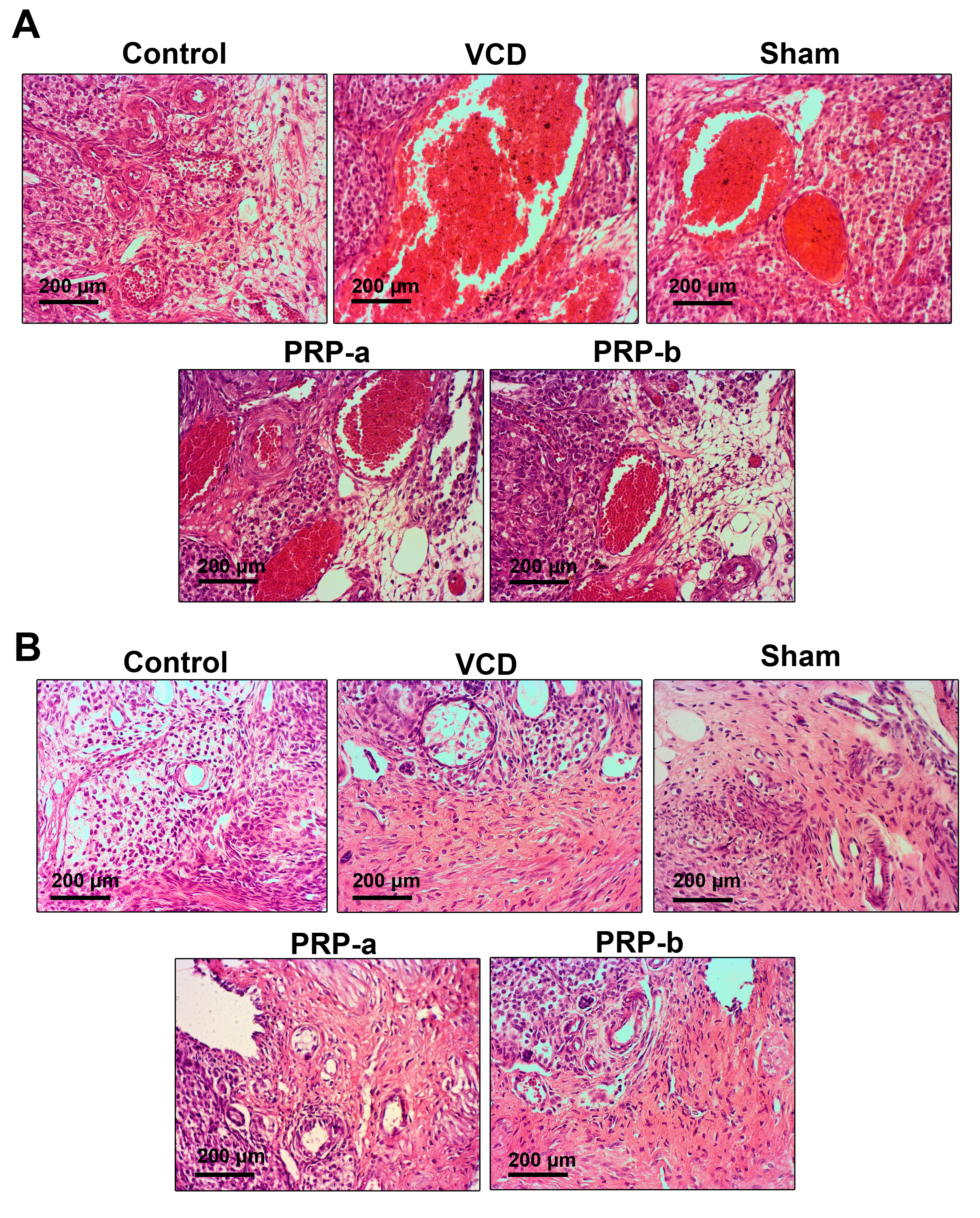

Supplement: Supplementary file 3 — Additional file 3: Supplementary Figure 3. Bright-field imaging after H&E staining to visualize hyperemia and vessel dilation (A), and fibrotic changes (B), following the administration of PRP in VCD-treated rats on 8th week. [file 12958_2020_638_MOESM3_ESM.tif]
