# Supplementary material for: Intra-ovarian injection of platelet-rich plasma into ovarian tissue promoted rejuvenation in the rat model of premature ovarian insufficiency and restored ovulation rate via angiogenesis modulation
Source: Reprod Biol Endocrinol. 2020 Aug 5;18:78. doi: 10.1186/s12958-020-00638-4 (PMC7405361; doi:10.1186/s12958-020-00638-4)
Supplement: Supplementary file 4 — Additional file 4: Supplementary Table 1. Mean follicle count in different groups after the intervention (mean ± SD). [file 12958_2020_638_MOESM4_ESM.docx]

**Supplementary Table 1.** Mean follicle count (the mean of all sampling points) in different groups after the intervention (mean±SD).

| Groups | Primary follicle | Secondary follicle | Antral follicle | Atretic primary follicle | Atretic secondary follicle | Atretic antral follicle |
| --- | --- | --- | --- | --- | --- | --- |
| Control | 3.92±3.304 | 9.17±5.253 | 9.08±4.228 | 2.67±2.919 | 4.08±2.347 | 1.50±0.577 |
| VCD | 0.00±0.000 | 0.00±0.000 | 0.00±0.000 | 15.67±18.370 | 10.13±3.113**^*^** | 3.25±2.217 |
| Sham | 0.00±0.000 | 0.00±0.000 | 0.00±0.000 | 10.67±7.328 | 6.75±3.696 | 2.92±1.912 |
| PRP-a | 2.33±1.587 | 1.92±0.833**^*^** | 1.79±1.150**^*^** | 4.92±2.409 | 1.38±1.417^†£^ | 0.75±0.739^†£^ |
| PRP-b | 2.71±2.413 | 5.00±0.544**^*^**^†£^ | 1.75±0.569**^*^** | 1.79±0.712^†^ | 3.29±1.022^†^ | 0.71±0.672^†£^ |

*Comparison between the control and other groups

†Comparison between the VCD and other groups

£Comparison between the Sham and other groups
